# Sloughing Prostatic Tissue, a Rare Complication Post Rezum Therapy: A Case Report

**DOI:** 10.7759/cureus.11722

**Published:** 2020-11-27

**Authors:** Ali Alothman, Rakan Alharbi, Ahmed Alasker, Yahya Ghazwani

**Affiliations:** 1 Urology, Ministry of the National Guard-Health Affairs, Riyadh, SAU; 2 Urology, King Abdullah International Medical Research Center, Riyadh, SAU; 3 Urology, King Saud bin Abdulaziz University for Health Sciences, Riyadh, SAU; 4 Medicine, King Saud bin Abdulaziz University for Health Sciences, Riyadh, SAU; 5 Medicine, King Abdullah International Medical Research Center, Riyadh, SAU

**Keywords:** rezum, bph, minimally invasive surgery, benign prostatic hyperplasia, luts, turp, lower urinary tract symptoms

## Abstract

Benign prostatic hyperplasia (BPH) is the most prevalent urological disease in men that leads to lower urinary tract symptoms (LUTS). The clinical presentation is, most likely, obstructive symptoms such as intermittency, hesitancy and poor stream, due to the obstructive nature of the pathology. BPH treatment approach varies. However, they can be divided into two main approaches which are non-surgical and surgical. Non-surgical methods usually started first, such as lifestyle modifications, watchful waiting, and medications. Hence, surgical intervention remains the mainstay of treatment to relieve clinical symptoms. Although transurethral resection of the prostate (TURP) is the gold standard, management is shifting towards minimally invasive surgeries such as Rezūm due to its good outcome and fewer adverse effects. We present a case of prostatic tissue sloughing, a rare complication post Rezūm system therapy in a 50-year-old male.

## Introduction

Benign prostatic hyperplasia (BPH) is the most prevalent urological disease in men that leads to lower urinary tract symptoms (LUTS). BPH incidence is in a proportional relationship with age. The clinical presentation is almost always obstructive since the majority of affected patients complain of obstructive voiding symptoms which account for approximately 25% in 55-year-old patients, and 50% in 70-year-old patients [1]. In Saudi Arabia, the presence of LUTS in 50 to 60-year-old patients reaches 52.2% and 30.6% in patients between 60 and 70 years of age [2]. BPH treatment approach varies. However, they can be divided into two main approaches which are surgical and non-surgical (conservative). Non-surgical methods usually started first, such as lifestyle modifications, watchful waiting, and medications. Hence, surgical intervention remains the mainstay of treatment to relieve clinical symptoms [3]. Different surgeries have been established for the treatment of BPH; nevertheless, monopolar transurethral resection of the prostate (TURP) has been superior to any minimally invasive surgery since 1970 [1,3]. However, TURP has its share of drawbacks which may render it less acceptable in some instances. TURP has a high rate of complications. For example, it may result in having retrograde ejaculation, erectile dysfunction, urethral stricture. Also, there may be a need of retreatment. Another example, it requires general or spinal anesthesia with mean hospital admission for two days [4]. New emerging minimally invasive techniques have been developed with similar outcomes as TURP but with fewer side effects [5]. One example: Rezūm system is a new minimally invasive thermal method that delivers a steam (vapor) via transurethral approach. It is considered advantageous due to its ability to result in a significant, prolonged symptomatic relief. Despite this, it is not free of complications. The majority of reported complications are not severe; the most common ones are dysuria, hematuria, hematospermia, symptoms of urgency, and urinary tract infections [6,7]. There are also rare major complications being reported such as prostate tissue sloughing, epididymo-orchitis, retrograde ejaculation, erectile dysfunction, bladder stone, and bladder neck contracture [8]. We report prostate tissue sloughing, a rare complication after Rezūm therapy which, to the best of our knowledge, was only reported twice in the English literature.

## Case presentation

**Figure 1:**
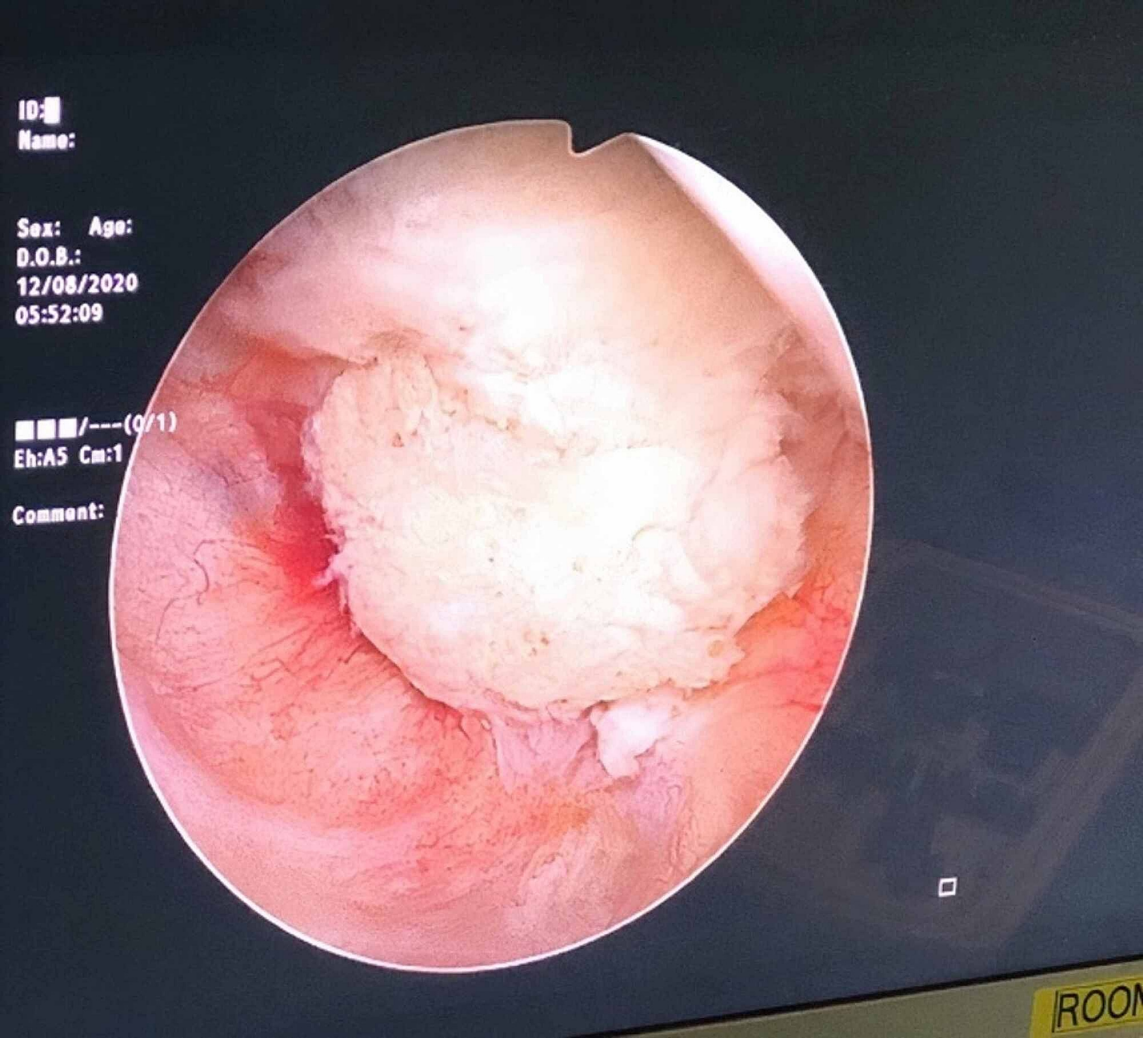
Sloughed tissue in the prostatic urethra

**Figure 2:**
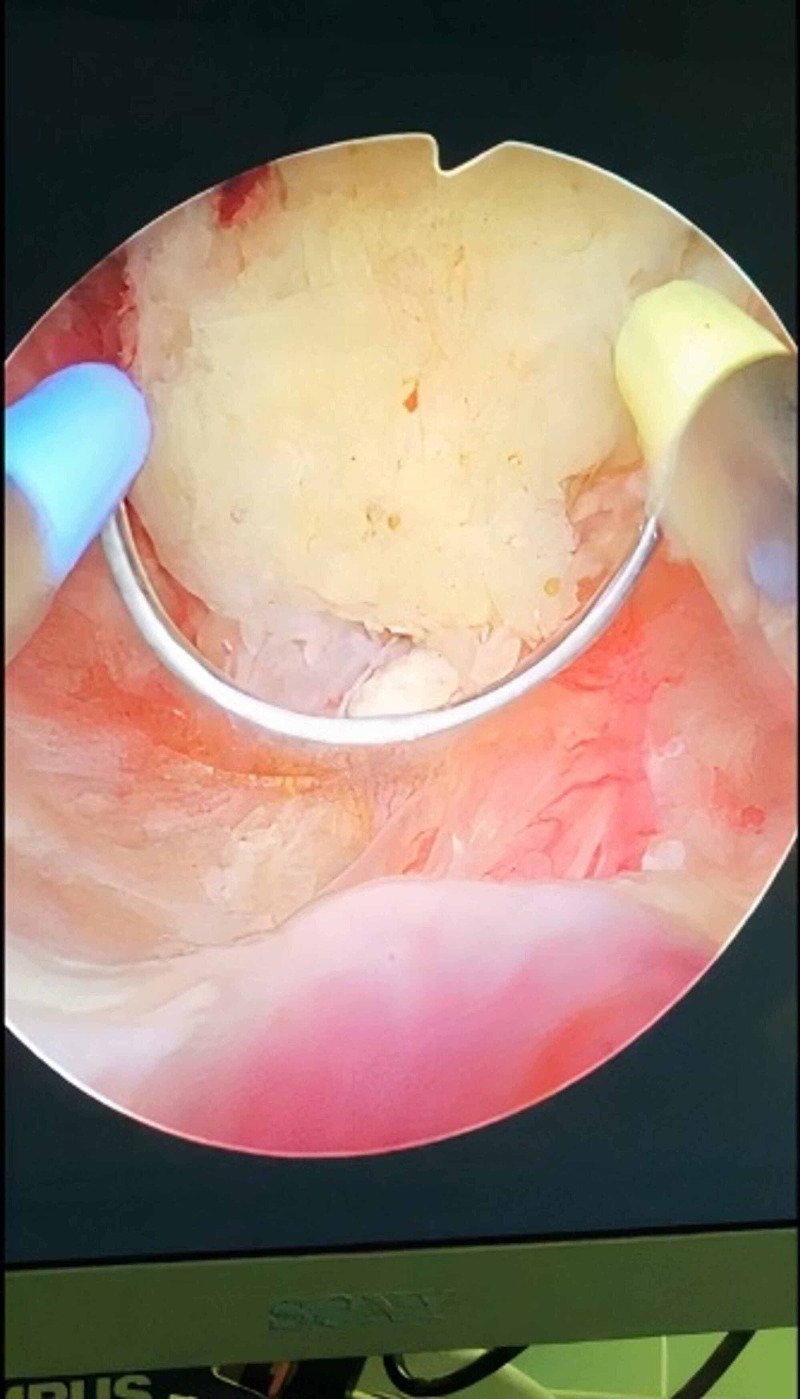
Necrotic tissue removed by resectoscope and electrocautery

**Figure 3:**
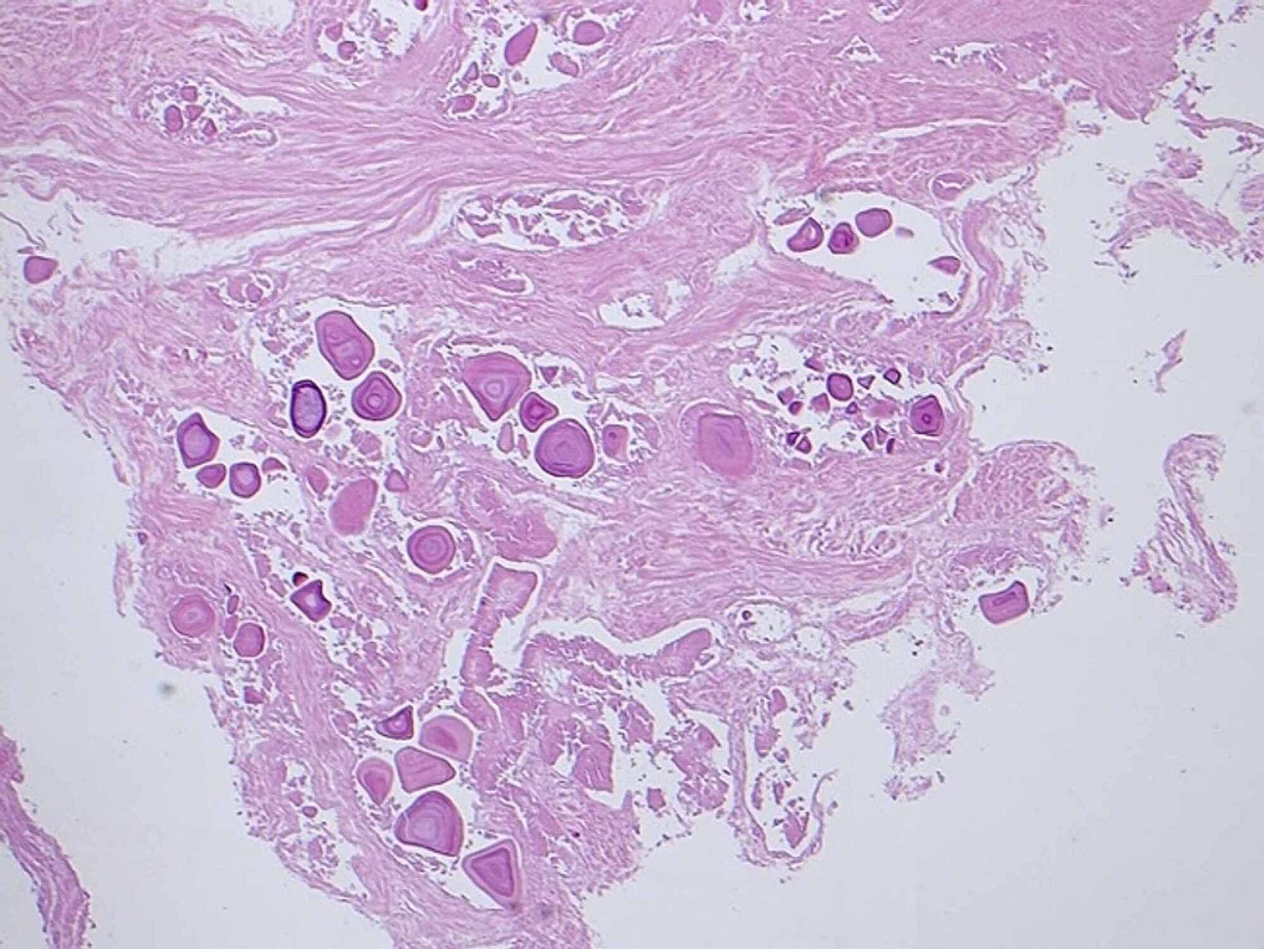
Cauterized and necrotic prostatic tissue with multiple corpora amylacea

A 50-year-old male with a known case of diabetes mellitus, hypertension, dyslipidemia, and BPH presented to our clinic complaining of frequency, nocturia, hesitancy, weak stream, and post micturition dribbling. He was following up in our clinic where he was started on tamsulosin, then dutasteride but with no improvement. The patient’s uroflowmetry peak flow was 11 milliliters per second (mL/s), Qmax was 14 mL/s, post-void residual urine was 70 mL, prostate-specific antigen was 0.68 nanograms per milliliter (ng/mL) and his prostate volume was 34.7 milliliters. The patient was offered Rezūm technique and was placed on size 18 Foley’s catheter. After four days, Foley’s catheter was removed, and the patient voided freely. The patient was seen in the clinic two weeks later and was complaining of severe dysuria and weak stream. Then, urinalysis and culture were ordered, and both were negative. The patient’s symptoms were relieved mildly with analgesics and he was given an appointment after three weeks for follow up. Later, the patient was offered cystoscopy. He was scoped and extensive sloughed tissue was found in the prostatic urethra, and the plan was to book him for TURP (Figure 1). TURP was done and necrotic tissue was removed by resectoscope and electrocautery (fulguration) was used for hemostasis (Figure 2). Subsequently, necrosed tissue was sent for histopathology (Figure 3). One day post-surgery, Foley’s catheter was removed, and the patient was discharged. After two weeks, the patient was followed up and was relieved of symptoms.

## Discussion

BPH is a common urological disease that often requires treatment. Although TURP is the gold standard, management is shifting towards minimally invasive surgeries such as Rezūm due to its good outcome and fewer adverse effects. The majority of reported Rezūm system’s adverse effects are mild in nature. The most reported complications are dysuria (16.9%), hematuria (11.8%), frequency and urgency (5.9%). These adverse events resolve either with routine treatment or without intervention in a couple of weeks of time [9]. On the other hand, there are a few rare major events reported after Rezūm therapy. For example, stone and bladder neck contracture were only reported once [8]. Also, prostate tissue sloughing is a rare complication that was only reported twice in the literature [8]. Major complications frequently require re-treatment with another surgical modality. In our case, the patient was scoped 50 days after complaining of LUTS and we found prostatic tissue sloughing which is a very rare complication. For that reason, the patient underwent TURP due to the rare complication and to relieve the patient’s symptoms. This case reports a rare complication to a new treatment modality that should be recognized by urologists.

## Conclusions

Management of BPH has been developing towards minimally invasive surgery due to the advancement in technologies and its availability. Rezum water therapy is a relatively new minimally invasive therapy for managing BPH. It has many reported advantages with mild complications. The major complications are not commonly reported or observed. For that reason, long term follow up for such cases is warranted and needs to be recognized by urologist for rare complications detection as it is a new method and there are limited data regarding its adverse effects.
